# Cultivating staff equality, diversity, and inclusion in higher education in the post-pandemic era: an organizational compassion perspective

**DOI:** 10.3389/fsoc.2024.1378665

**Published:** 2024-05-30

**Authors:** Haleh Hashemi Toroghi, Fiona Denney, Ace Volkmann Simpson

**Affiliations:** ^1^Brunel Business School, College of Business, Arts and Social Sciences, Brunel University London, Uxbridge, United Kingdom; ^2^Center for Compassion Studies, New York, NY, United States

**Keywords:** organizational compassion theory, equality, diversity, inclusion, higher education, wellbeing, suffering, COVID-19

## Abstract

The COVID-19 pandemic has exacerbated pre-existing challenges faced by academic staff in UK higher education and drawn attention to issues of Equality, Diversity, and Inclusion (EDI). Amidst global competitiveness and workplace pressures, challenges such as managerialism, increased workload, and inequalities have worsened, significantly impacting mental health. This paper presents a conceptual analysis connecting EDI with organizational compassion within the context of Higher Education. The prioritization of organizational compassion is presented as a means to enhance sensitivity to EDI in the reconstruction of post-pandemic learning environments. Anchored in the organizational compassion theory and the NEAR Mechanisms Model, our study contributes to the intersection of the organizational compassion, EDI and higher education literatures by exploring how fostering compassion relations can contribute to enhancing EDI. This offers a new perspective to creating a more humane and supportive higher education environment.

## Introduction

The COVID-19 pandemic has significantly impacted higher education, as evidenced by several studies (Pan, 2020; Rapanta et al., 2020; Dinu et al., 2021; Wray and Kinman, 2022; Denney, 2023), intensifying pre-existing challenges such as managerialism, increased workload, and inequalities, all of which have worsened the mental health of academic staff. As the pandemic reshapes the higher education environment, it accentuates critical areas requiring attention. One such area relates to Equality, Diversity, and Inclusion (EDI), concerned with the ensuring fair treatment, access, opportunity, and advancement for all, including those of minority ethnic and identity groups (Nishii, 2013; Górska et al., 2021; Alkan et al., 2022; Mickey et al., 2023).

In response to the challenges heightened by the COVID-19 pandemic, UK universities have been compelled to re-evaluate their teaching methods and support systems. Unfortunately, these changes have inadvertently burdened academic staff, leading to heightened work-related stress and additional difficulties, particularly for staff of color and women (Górska et al., 2021; Mickey et al., 2023). The amalgamation of increased demands to prioritize a 'students first' logic (Denney, 2022) and a simultaneous reduction in available resources has taken a considerable toll on the overall well-being of academic staff (Wray and Kinman, 2022).

The UK's higher education system finds itself amidst upheaval, as it navigates a global atmosphere of competitiveness, complexity, and uncertainty (Maratos et al., 2019; Denney, 2020, 2021b, 2023; Waddington, 2021). Factors such as increasing market competition, managerialism, workplace inequality, and continuous workload pressure have contributed to heightened mental health risks among academic staff (Kinman, 2001; Wallmark et al., 2013; Waddington, 2016; Denney, 2020; Urbina-Garcia, 2020; Shen and Slater, 2021; D'Cruz et al., 2023). Particularly vulnerable to such pressures are those staff associated underrepresented minority groups such as women facing gender-related challenges; LGBTQ+ individuals encountering visibility and acceptance issues, individuals with disabilities facing accessibility and attitudinal barriers, international scholars navigating cultural adjustments; religious minorities contending with potential bias; and individuals from Black, Indigenous, and People of Color (BIPOC) communities.

Considering these challenges, the significance of EDI in higher education cannot be overstated. EDI represents foundational values that advocate for the equitable treatment and full engagement of all individuals, particularly those historically marginalized or subjected to discrimination based on factors such as gender, background, identity, and (dis)ability (Gill et al., 2018; Özbilgin, 2019). This commitment extends to addressing the specific barriers encountered by various marginalized populations, including people of color, women, LGBTQ+ individuals, individuals with disabilities, indigenous peoples, refugees, and those on the autism spectrum (Wolbring and Lillywhite, 2021). Despite historical efforts to advance EDI in higher education, academic staff still grapple with inequality, discrimination, and exclusion events (Özbilgin, 2009; Fossland and Habti, 2022; Hofstra et al., 2022). Recognizing that cultivating EDI is of core importance in higher education, there is a critical need to reconstruct a compassionate and inclusive learning environment, extending beyond embracing diversity among academic staff.

To address these challenges, our conceptual analysis explores the intersection of these concepts and issues through reflective practices. We propose leveraging an organizational compassion lens. Within the higher education context, compassion can be understood as a dynamic interpersonal and social process characterized by NEAR: noticing suffering experienced by students and academic staff, empathizing with their distress, appraising their suffering through a specific perspective, and responding by undertaking meaningful actions to alleviate their hardships (Kanov et al., 2004; Dutton et al., 2014; Worline and Dutton, 2017, 2022; Simpson et al., 2019a). Investigating the dynamic interaction between workplace compassion and the advancement of EDI, along with their reciprocal impacts, emerges as a recognized potential focus within the field of applied compassion scholarship. This holds particular significance considering the criticism surrounding the efficacy of diversity training, a prevalent tool in EDI initiatives, often deemed ineffective or even counterproductive (Dobbin and Kalev, 2018).

Exploration of the nexus between organizational compassion and EDI is, however, still at an early stage of emergence. Gibbs (2019, p. 161) argues that “compassion lies at the core of diversity.” Emirza (2022, p. 31) suggests that specifically compassionate leadership can act as a mechanism for “fostering a sense of inclusion among diverse employees.” Kizilenis Ulusman et al. (2023, p. 22), drawing insights from their empirical study involving 25 female migrant participants, propose that “compassion in organizations can be a valuable resource for addressing diversity-related challenges.” These observations indicate the potential of approaching EDI through an organizational compassion lens. Accordingly, in this article we seek to theorize further the potential of promoting EDI through an organizational compassion theory lens. More specifically we propose the NEAR (Noticing, Empathizing, Assessing, and Responding) Mechanisms Model as practical framework to guide the cultivation of EDI in higher education. This strategic approach aims to support not only alleviating the struggles faced by marginalized academic staff but to build a more human-centered and compassionate higher education environment in the post-pandemic era.

Our paper is structured as follows: firstly, we provide a comprehensive exploration of the impact of neoliberal managerialism on higher education, its challenges to EDI, the associated mental health impacts. Additionally, we propose a framework for rebuilding learning environments through organizational compassion.

## The impact of neoliberal managerialism on higher education EDI

Universities are traditionally envisioned as learning environments that serve to create a positive and safe foundation for teaching and are integral elements of the education system. Leadership within universities assumes a vital role in promoting the development of positive and inclusive environments in which knowledge creation, cultural transmission, free thought, and the pursuit of truth can thrive, as well as inspiring the values of integrity, respect, and compassion throughout their institutions (Flückiger, 2021; Waddington, 2021). Additionally, universities are committed to fostering equality and inclusion among diverse groups of individuals (Özbilgin and Erbil, 2021, 2023). While universities often express commitment, particularly for staff members, including academic staff or students, the reality may not always reflect this dedication (Maratos et al., 2019). Moreover, as Marginson (2020) highlights, higher education faces fundamental challenges associated with a growing economy and social inequality globally. He emphasizes that while higher education plays a vital role, it cannot address these challenges thoroughly on its own. Other sectors, such as wage and salary determination, taxation, and government programmes are often more influential in shaping societal inequalities (Marginson, 2020). This broader view is crucial when exploring into the impact of neoliberal managerialism on EDI in higher education, as we will further explore next.

### Neoliberal managerialism undermining EDI in higher education

The impact of neoliberal managerialism on EDI in higher education has become more evident as the landscape has undergone significant changes (Maratos et al., 2019; Denney, 2020; Waddington, 2021). This shift has created a global atmosphere of competitiveness, complexity, and uncertainty, particularly in the UK, where higher education is in a perpetual state of change and turbulence (Denney, 2021b). Neoliberalism's influence on higher education in the UK dates back to the 1970s, coinciding with the political leadership of Margaret Thatcher and Ronald Reagan and the subsequent global spread of four key changes in political economy capitalism: privatization, deregulation, financialization, and globalization (Radice, 2013). This neoliberal paradigm, as highlighted by Benatar et al. (2018), is associated with negative consequences such as increased poverty, inequality, and the pervasive commercialization of social life and educational systems. Radice (2013) contends that neoliberalism has manifested as new managerialism in the UK public sector, characterized by the adoption of private business sector structures, technologies, and values. Specifically, new managerialism blends hierarchical control with elements of the free market, imposing private sector values on the public sector (Deem, 1998) and shifting the university's focus from elite education to contributing marketable skills and research outputs to the “knowledge economy” (Radice, 2013, p. 408). New managerialism in higher education involves internal cost centers, staff rivalry, marketization of public sector services, and the monitoring of efficiency through outcome measurement and performance evaluation, particularly for academic staff (Deem, 1998). Performativity in managing academic labor in UK universities is a key aspect of new managerialism, as detailed performance measurement shifts the culture from collegial to managerial (Cowen, 1996).

### EDI challenges faced by academics

Academic women face unique challenges as a result of cultural shifts in higher education. UK higher education institutions' underlying systems, structures, processes, and cultures are inherently referred to as masculine managerialism since they were designed for men, contributing to systemic gender biases. The combination of masculinized higher education institutions with neoliberalism's performativity has created a challenging environment hindering the progression of academic women into senior leadership roles (Denney, 2021a).

In tandem with gender inequalities, racial disparities persist in higher education, posing a significant concern for academia. Fossland and Habti (2022) highlight ongoing racial inequalities in higher education, emphasizing the continued support for white male scientists and their scientific contributions, diminishing the visibility of research contributions from women and minority scholars. This intersectionality of gender and race underscores the need for a comprehensive approach to address multiple dimensions of inequality in academia.

It is crucial to recognize and rectify inequality issues in UK higher education institutions across various other dimensions. LGBTQ+ representation, disability inclusion, and the intersectionality of identities must also be central considerations in fostering a truly inclusive higher education environment. Research consistently demonstrates that diverse and inclusive institutions not only enhance innovation and research but also contribute to advancements in science (Østergaard et al., 2011; Nielsen et al., 2017, 2018). Thus, a comprehensive approach to EDI is essential for the flourishing of academics representing diverse groups in higher education, but also the realization of new discoveries and advancements in knowledge.

### Mental health and other impacts

Considering the EDI challenges faced by academic staff in UK higher education institutions, it is not surprising that many academics struggle in what are experienced as challenging and toxic environment in which to work (Denney, 2020). This is notably reflected in highlights from the latest Times Higher Education University Workplace survey (2016), which included 1,398 (49 percent) UK academics from nearly 150 universities across the UK (as shown in Table 1), revealing that academics are increasingly suffering at work. Consideration of these pre-pandemic findings would suggest that, in the interim, circumstances have likely deteriorated further.^1^

**Table 1:** Times Higher Education University Workplace Survey 2016 (Grove, 2016).

Academics express a high level of stress and dissatisfaction with the poor leadership of both their department and their institution, and a significant percentage are considering leaving their current position.
54% of academics feel their voices are not heard by their university leadership.
Job insecurity is one of the major concerns for many academics, with 40% of respondents believing their job is not secured.
Academics are concerned about three main issues: growing managerialism and its associated marketizations and rankings-driven policies; frequent performance measurement, and objective setting; increasing bureaucratic system and standardization that diminishes professional discretion.
Half of the academics are concerned about redundancies related to metrics-based performance measures and feel anxieties and pressure.
Most academics feel overworked and burnout.
There are some concerns amongst academics about the condition of their workplace bullying, discrimination, and harassment.

As illustrated in Table 1, academics reported experiencing high levels of stress and dissatisfaction with their institutions' leadership, with a significant percentage considering resigning from their roles. Additionally, 54% of academics felt unheard by their institution's leadership (Grove, 2016). These findings are concerning and could be indicative of a dearth of compassionate leadership at UK universities. The survey further revealed that a substantial number of academics were worried about job insecurity, with 40% feeling their positions were at risk of redundancy. Specifically, academics expressed anxiety and pressure related to metrics-based performance measures, as well as concerns about expanding managerialism, frequent performance monitoring, and an increasingly bureaucratic system that eroded professional discretion (Grove, 2016).

A more recent empirical study surveying over five thousand academic staff from across the UK found that academic personnel frequently encountered difficult, stressful, and sometimes humiliating situations (Erickson et al., 2021). More relevant to the state of EDI in UK higher education institutions, this study highlighted those chronic issues of bullying, discrimination, and harassment, along with elevated levels of mental health difficulties, general health and wellbeing concerns, and alarming levels of hopelessness and dissatisfaction among UK academics. Other research has reported that disabled university professors, instructors, teachers, and researchers, were particularly vulnerable to unfair treatment, biased discrimination, bullying, and harassment, placing them at a higher risk of burnout (Wolbring and Lillywhite, 2023).

In sum, the pervasive impact of neoliberal managerialist logic on academic life, as evidenced by the stress, dissatisfaction, and mental distress experienced by academic staff, particularly those in vulnerable positions, underscores the urgency of transformative measures. The existing conditions, marked by limited EDI and the resulting toxic work environment in universities, demand a paradigm shift toward organizational compassion. The distress faced by academic members not only highlights the need for immediate alleviation but also emphasizes the imperative to rebuild universities with a foundation rooted in compassion. Beyond addressing the sufferings of academic staff, this transformation calls for a steadfast commitment to respecting diversity, fostering inclusion, and valuing equality, as emphasized by Gibbs (2019). In the upcoming section, we consider the value of compassion and its pivotal role in promoting EDI in the context of higher education.

## Re-building learning environments through organizational compassion: a theoretical framework for promoting EDI in higher education

Scholars have recently begun to emphasize the value of organizational compassion and caring in advancing EDI (Rynes et al., 2012), which is also applicable to the higher education context. Compassion serves as a dynamic force that bridges individuals with the wider community and forms the core principles for EDI (Nussbaum, 1996; Gibbs, 2019). Expanding on this viewpoint, Waddington (2018) argues that universities have a moral and legal responsibility to take reasonable measures to protect all individuals associated with the institutions from personal physical and/or emotional suffering, including both academic staff and students.

Organizational scholars view compassion not merely as an emotion but as a dynamic interpersonal and social process (Goetz et al., 2010; Simpson et al., 2019b, 2022). In the context of higher education and EDI it begins with recognizing suffering among students and academic staff, particularly within areas of inequality, injustice, and racism. Beyond recognizing suffering, the compassion process continues when there is empathy for the sufferer's pain, assessing of the suffering through a particular lens, and a response of taking meaningful action to alleviate the distress (Kanov et al., 2004; Dutton et al., 2014; Strauss et al., 2016; Stellar et al., 2017; Worline and Dutton, 2017, 2022; Anstiss et al., 2020; Waddington, 2021).

**Figure 1:**
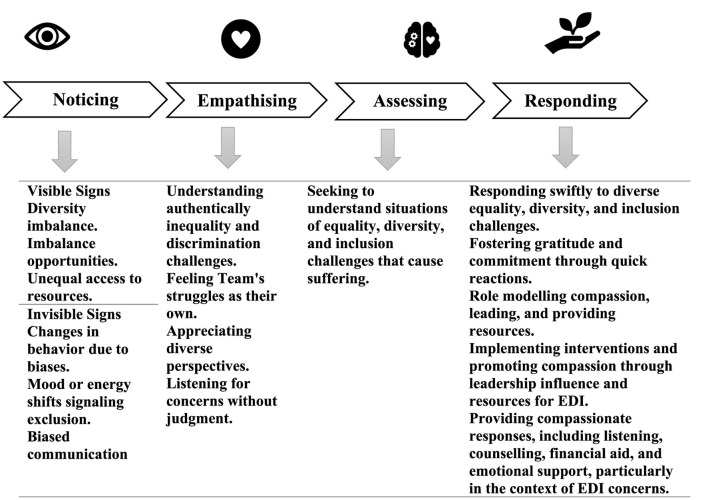
NEAR framework lens (Dutton et al., 2014; Simpson and Farr-Wharton, 2017; Simpson et al., 2019a, 2023).

Building on this definitional foundation of organizational compassion as a NEAR process of noticing, empathizing, appraising and responding to address workplace suffering, Simpson and Farr-Wharton (2017), Simpson et al.'s (2019b, 2020) NEAR mechanisms model of organizational compassion integrates these key processes with facilitative organizational mechanisms. We argue that this model, which offers a practical framework for cultivating organizational compassion, can be drawn upon to manage organizational processes fostering EDI systematically and consciously (Figure 1). ^2^

### Noticing

In the context of fostering EDI within higher education, an essential initial step in fostering healing, is the act of noticing distress. Compassionate leaders play a crucial role in fostering an environment where academic members can openly discuss their distress. By cultivating a culture openness and trust, leaders can inspire employees to support one another through compassionate actions whether through words, presence, or in providing tangible support, such as making available existing resources or coordinating to generate new resources. Such empowerment leads to the cultivation of a collective capacity for compassion, which is crucial during difficult times. These examples indicate that compassionate leadership involves more than showing personal compassion and caring for a colleague or dependent in need (Dutton et al., 2002; Poorkavoos, 2016).

In the context of addressing inequality within the academic setting, noticing becomes the initial step in cultivating awareness of indicators, both visible and invisible (Simpson et al., 2015). Recognizing these signals is crucial for noticing inequality in the academic environment. These subtle signals can demonstrate through changes in mood, energy levels, daily routines, language use, or changes in behavior (Dutton et al., 2014).

In the realm of higher education not everyone openly articulates their struggles. This highlights the importance of leaders being sensitive to these less overt signs. Noticing these subtle signals enables leaders to identify potential imbalance opportunities, access to resources, or instances of bias. A deeper awareness of these subtle indicators empowers leaders to take proactive action, cultivating a more fair, equitable and inclusive learning environment for all (Chang and Milkman, 2020; Özbilgin and Erbil, 2023).

### Empathizing

Empathizing is the second step in the NEAR framework. In the pursuit of equality, a leader actively engages in this crucial step by authentically connecting with the challenges faced by their followers, internalizing the team's struggles as if they were their own (Dutton et al., 2014; West, 2021). This empathetic approach, closely connected with recognizing and appreciating each team member's unique perspective, fosters an environment where attentive listening and mutual understanding flourish (Gibbs, 2019).

### Appraising

Appraising is the third step which refers to leaders keenly assess the situation and underlying causes of challenging work situations that contribute to academic staff suffering, demonstrating a keen appraisal of their team's struggles (West, 2019, 2021). In the context of promoting equality, diversity, and inclusion, this proactive assessment aims to identify and rectify any inequalities, job segregation, unfairness, or discriminatory practices that may be triggering difficulties for team members (DiTomaso and Parks-Yancy, 2014). Furthermore, it involves establishing a socially sustainable learning environment (D'Cruz et al., 2023) where individuals are treated fairly and have equal opportunities. This includes addressing issues related to gender inequality and ensuring that every team member, regardless of background, can thrive and contribute to their fullest potential.

### Responding

Thus far, we have presented the NEA (noticing, empathizing, and appraising) process model of organizational compassion theory. However, a response is required to complete the process (Simpson et al., 2020). Responding involves taking action to address challenges faced by a diverse group of academic members (Dutton et al., 2006; Emirza, 2022). A compassionate response particularly involves recognizing a co-worker's suffering and providing resources to alleviate it. When the response to alleviate suffering is prompt, it indicates efficient compassion organizing to address diverse needs and promote an inclusive environment (Dutton et al., 2002, 2006; Worline and Dutton, 2017). A quick reaction to suffering is seen as a hallmark of genuine care and concern, enhancing the likelihood of gratitude and commitment from the recipient of compassion (Simpson et al., 2013, 2015).

Thus far we have looked at an organizational compassion informed view of how EDI can be cultivated within higher education institutions through a fourfold NEAR process, however, it is also important to consider how this process can be facilitated through leveraging organizational mechanisms. In the following section, we focus specially on the mechanisms of organizational leadership and organizational culture.

### Organizational mechanisms of leadership and culture

Among the six organizational social architecture mechanisms crucial for facilitating workplace compassion, such as relational networks, routine practices, roles, and stories told, leadership and culture stand out as pivotal factors that compassionate organizations can prioritize to enhance their compassion capabilities (Dutton et al., 2006, 2014; Worline and Dutton, 2017). Accordingly, we will discuss leadership and culture in turn within the context of leveraging organizational compassion to enhance EDI.

### Leadership

Leaders in higher education have a duty to not only uphold certain shared values such as a passion and commitment to the pursuit of the truth, knowledge dissemination, and freedom (Dearing and National Committee of Inquiry Into Higher Education, 1997). Additionally, they are responsible for promoting an inclusive environment where all members, such as academic members and students, feel welcomed, valued, and respected regardless of their identities, backgrounds, and experiences (Emirza, 2022). These fundamental values provide an opportunity for those who work in higher education, particularly academic members, to engage more to community and teaching activities for the benefit of all (Waddington, 2021). Therefore, universities would benefit from cultivating a compassionate caring working atmosphere, with leaders playing a significant role. It is observed, however, that this is often not the case, especially for academics (Maratos et al., 2019), particularly those representing EDI domains.

Research findings show that traditional equality, diversity, and inclusion training within institutions have faced criticism for being ineffective or even having some negative effects (Dobbin and Kalev, 2018). Furthermore, universities' hectic and toxic work environments significantly impact the physical health, emotional wellbeing, meaningful social connections, and cognitive performance of academic members (Waddington, 2016; Denney, 2020, 2021a). Despite the growing stress among academic members at work, universities have the potential to be compassionate caring environments for all academic staff and students (Waddington, 2016, 2021).

Within the context of suffering and EDI, leaders can start the healing process by role modeling compassionate behavior through their presence, leading processes of sense making, and providing resources to take action in addressing distress (Dutton et al., 2002, 2006; Worline and Dutton, 2017). Furthermore, leaders have the resources and influence to promote compassion and make meaning in the institutions they lead (Dutton et al., 2006; Worline and Dutton, 2017; Simpson et al., 2019a, 2020, 2022). Compassionate responses may involve attention from leaders, empathic listening (Worline and Dutton, 2017), counseling and psychological support, financial aid (Simpson et al., 2019a, 2020), compassionate leave, and hybrid working during times of crisis, such as COVID-19. These compassionate leadership interventions can enhance inclusiveness and support diversity (Emirza, 2022).

An argument for positing that leadership and inclusive culture are perhaps the most important organizational compassion mechanisms for rebuilding learning environments to align with the principles of EDI, is that leaders significantly influence the extent to which inclusive culture mechanism may be deployed or undermined (see Table 2). Accordingly, we next discuss culture.

**Table 2:** Impact of compassionate leadership and culture on promoting EDI.

Positive physical atmosphere	Compassionate values reflected in the physical environment of institutions.
	Architecture of university buildings, interior design and more contribute to a supportive atmosphere.
Humanistic values	Compassionate cultures promote values such as respect, inclusiveness, fairness, and dignity.
	Espoused values align with compassionate principles such as organizational strategies.
Influence on interpretation and action	Basic assumptions shape generous interpretation and compassionate action.
	Compassionate leaders play a key role in shaping espoused values and basic assumptions.
Fostering inherent value and deservedness	Compassionate cultures highlight the inherent value of all humans.
	Members act compassionately based on these beliefs.
Leadership and culture alignment	Compassionate leadership goes hand in hand with culture development.
	Compassionate leaders significantly impact on a compassionate institutional culture.
Promoting EDI through leadership	Compassionate leadership supports EDI through inclusive behavior.
	Compassionate leadership influences structures and norms for a diverse and inclusive culture.

### Organizational culture

Culture is an important explanatory organizational concept (Schein, 2010) that refers to the combination of the members' shared patterns of meaning, beliefs, attitudes, and practices (Simpson et al., 2019a, 2020). It can be observed at three levels: artifacts, espoused values, and basic assumptions. Artifacts include visible organizational structures and processes such as an institution's physical environment, the architecture of university buildings, interior design, landscapes, technologies, and uniforms. Espoused values include organizational' strategies, goals, and philosophies such as value statements, code of conduct, mission statements. Basic assumptions are the unconscious, taken for granted beliefs, values, and feelings that underpin an organization's culture (Schein, 2010). Level one, artifacts, is the most visible. Level two, espoused values, and beliefs are found in published organizational statements on websites, annual reports, policy documents and training material. Level three, basic assumptions, are the most difficult to observe and define (Schein, 2010).

Compassionate leaders can play a significant role in shaping espoused values and basic assumptions, which are critical aspects of a university's culture that influence compassion competence. When compassionate cultures promote the inherent value, capability, and deservingness of all humans, members are more likely to interpret pain generously and engage in compassionate action. Organizational cultures that support compassion competence are characterized by humanistic values such as respect, teamwork, collaboration, inclusiveness, stewardship, dignity, and fairness. To promote a compassionate organizational culture, compassionate leaders should articulate and support values, beliefs, and norms that support human wellbeing, dignity, respect, and inclusion for all members of the academic community (Worline and Dutton, 2017; Gibbs, 2019; Emirza, 2022). As Schein (2010) noted, leadership and culture often go hand in hand, and compassionate leadership can have a significant impact on developing a compassionate institutional culture. Compassionate leadership can promote EDI and shape the culture of their university through their leadership behavior and the structures, routines, rules, and norms that they help to implement individually and collectively (Schein, 2010; West, 2021).

Research findings show that cultivating organizational compassion has numerous benefits for both individuals and organizations (Simpson et al., 2020). These include higher levels of positive emotions, employee loyalty, affective commitment within an organization, and high-quality connection among members of the organization (Lilius et al., 2008, 2011) along with a strengthened sense of authenticity (Ko and Choi, 2020). Consequently, organizational compassion practices hold much promise for alleviating suffering and addressing high levels of staff burnout, anxiety and turnover (Simpson et al., 2020), particularly within the higher education sector. This aligns with the values of equality, diversity, and inclusion, contributing to a more compassionate and caring working environment for staff and learning environment for students.

## Discussion and call to action

In the face of current challenges in higher education, prioritizing EDI together with compassion and humanity is crucial for alleviating much suffering experienced by those working within the sector (Gibbs, 2019; Özbilgin, 2019). The challenges faced by academic staff in UK higher education, exacerbated by the COVID-19 pandemic, demand an holistic and compassionate response. By incorporating reflective practices into our analysis, we aim to reflect the voices and experiences of academic staff, amplifying their perspectives in our exploration of EDI challenges. Prioritizing EDI through the cultivation of workplace compassion provides an opportunity for rebuilding inclusive learning and working environments post-pandemic. Our conceptual analysis suggests that at the intersection of compassion, EDI, and higher education the application of the NEAR Mechanisms Model, rooted in organizational compassion theory, offers a practical framework for leaders to navigate the complexities of EDI challenges in higher education.

A commitment to EDI combined with a compassionate organizational approach goes beyond merely addressing immediate challenges. It lays the foundation for a sustainable and inclusive future in higher education. By fostering an environment that values diverse perspectives and promotes equal opportunities, institutions can empower individuals and contribute to long-term systemic change. This not only benefits the current generation of academic professionals but also sets a precedent for future cohorts, creating a more resilient and adaptable educational landscape. In essence, the integration of EDI and compassion serves as a transformative force that extends far beyond the current exigencies, shaping a more equitable and compassionate academic community for generations to come.

## Author contributions

HHT: Writing—original draft. FD: Supervision, Writing—review & editing. AS: Supervision, Writing—review & editing.

## Footnotes

^1^Although the reference gives information about all staff in HE, our table here only refers to academics.

^2^We have brought together existing compassion process models and applied them to EDI to create this new framework.

## References

[B1] Alkan D. P. Ozbilgin M. Kamasak R. (2022). Social innovation in managing diversity: COVID-19 as a catalyst for change. Eq. Div. Inclusion Int. J. 41, 709–725. 10.1108/EDI-07-2021-0171

[B2] Anstiss T. Passmore J. Gilbert H. (2020). Compassion: the essential orientation. The Psychol. 33, 38–42.

[B3] Benatar S. Upshur R. Gill S. (2018). Understanding the relationship between ethics, neoliberalism and power as a step towards improving the health of people and our planet. Anthropocene Rev. 5, 155–176.

[B4] Chang E. H. Milkman K. L. (2020). Improving decisions that affect gender equality in the workplace. Org. Dyn. 49:100709. 10.1016/j.orgdyn.2019.03.00236612341

[B5] Cowen R. (1996). Performativity, post-modernity and the university. Comp. Educ. 32, 245–258. 10.1080/0305006962887620521009

[B6] D'Cruz P. Kanov J. Simpson A. V. Noronha E. Dodson S. Pei A.. (2023). “Leveraging compassion to address inequality at work,” in Academy of Management Annual Meeting Proceedings, 10–11. 10.5465/AMPROC.2023.13561symposium

[B7] Dearing R. National Committee of Inquiry Into Higher Education (1997). Higher Education in the Learning Society: Report of the National Committee. London: NCIHE, 34.

[B8] Deem R. (1998). New managerialism and higher education: the management of performances and cultures. in in universities in the United Kingdom. Int. Stud. Sociol. Educ. 8:14. 10.1080/0962021980020014

[B9] Denney F. (2020). Compassion in higher education leadership: casualty or companion during the era of coronovirus? johepal. 1, 41–47. 10.29252/johepal.1.2.41

[B10] Denney F. (2021a). A glass classroom? The experiences and identities of third space women leading educational change in research-intensive universities in the UK. Educ. Manage. Admin. Leadership 22:17411432211042882. 10.1177/17411432211042882

[B11] Denney F. (2021b). The “golden braid” model: courage, compassion and resilience in higher education leadership. johepal. 2, 37–49. 10.52547/johepal.2.2.37

[B12] Denney F. (2022). Working in universities through COVID-19: an interpretation using the lens of institutional logics. in academy of management proceedings. Acad. Manage. 10510:12431. 10.5465/AMBPP.2022.12431abstract

[B13] Denney F. (2023). “Get on with it. cope.”: the compassion-experience during COVID-19 in UK universities. Front. Psychol. 14:2404. 10.3389/fpsyg.2023.1112076 37416541 PMC10321556

[B14] Dinu L. M. Dommett E. J. Baykoca A. Mehta K. J. Everett S. Foster J. L. H.. (2021). A case study investigating mental wellbeing of university academics during the COVID-19 pandemic. Educ. Sci. 11:702. 10.3390/educsci11110702

[B15] DiTomaso N. Parks-Yancy R. (2014). The social psychology of inequality at work: Individual, group, and organizational dimensions. Handb. Soc. Psychol. Ineq. 12, 437–457. 10.1007/978-94-017-9002-4_18

[B16] Dobbin F. Kalev A. (2018). Why doesn't diversity training work? The challenge for industry and academia. Anthropo. Now 10, 48–55. 10.1080/19428200.2018.1493182

[B17] Dutton J. E. Frost P. J. Worline M. C. Lilius J. M. Kanov J. M. (2002). Leading in times of trauma. Harvard Bus. Rev. 11, 54–61. 12964467

[B18] Dutton J. E. Workman K. M. Hardin A. E. (2014). Compassion at work. Ann. Rev. Org. Psychol. Org. Behav. 1, 277–304. 10.1146/annurev-orgpsych-031413-091221

[B19] Dutton J. E. Worline M. C. Frost J. Lilius J. (2006). Explaining compassion organizing. Admin. Sci. Q. 51, 59–96. 10.2189/asqu.51.1.5921821037

[B20] Emirza S. (2022). “Compassion and diversity: a conceptual analysis of the role of compassionate leadership in fostering inclusion,” in Leading With Diversity, Equity and Inclusion: Approaches, Practices and Cases for Integral Leadership Strategy, eds. J. Marques, and S. Dhiman (Cham: Springer), 31–46. 10.1007/978-3-030-95652-3_3

[B21] Erickson M. Hanna P. Walker C. (2021). The UK higher education senior management survey: a statactivist response to managerialist governance. Stu. Higher Educ. 46, 2134–2151. 10.1080/03075079.2020.1712693

[B22] Flückiger Y. (2021). The conditions for higher education institutions to meet the social challenges ahead. johepal. 2, 120–129. 10.52547/johepal.2.1.120

[B23] Fossland T. Habti D. (2022). University practices in an age of supercomplexity: revisiting diversity, equality, and inclusion in higher education. J. Praxis Higher Educ. 4, 1–10. 10.47989/kpdc355

[B24] Gibbs P. (2019). At the core of diversity is compassion. Glob. Div. Manage. Fusion Ideas Stories Prac. 2019, 161–171. 10.1007/978-3-030-19523-6_15

[B25] Gill G. K. McNally M. J. Berman V. (2018). Effective Diversity, Equity, and Inclusion Practices. Healthcare Management Forum. Los Angeles, CA: SAGE Publications, 196–199. 10.1177/0840470418773785 30114938

[B26] Goetz J. L. Keltner D. Simon-Thomas E. (2010). Compassion: an evolutionary analysis and empirical review. Psychol. Bullet. 136:351. 10.1037/a0018807 20438142 PMC2864937

[B27] Górska A. M. Kulicka K. Staniszewska Z. Dobija D. (2021). Deepening inequalities: What did COVID-19 reveal about the gendered nature of academic work?. Gender Work Org. 28, 1546–1561. 10.1111/gwao.12696 34219993 PMC8239576

[B28] Grove J. (2016). THE University Workplace Survey 2016: results and analysis. Times Higher Education (THE), 1–33. Available online at: https://www.timeshighereducation.com/features/university-workplace-survey-2016-results-and-analysis

[B29] Hofstra B. McFarland D. A. Smith S. Jurgens D. (2022). Diversifying the professoriate. Socius 8:23780231221085120. 10.1177/23780231221085118

[B30] Kanov J. M. Maitlis S. Worline M. C. Dutton J. E. Frost P. J. Lilius J. M. (2004). Compassion in organizational life. Am. Behav. Sci. 47, 808–827. 10.1177/0002764203260211

[B31] Kinman G. (2001). Pressure Points: A Review of Research on Stressors and Strains in UK Academics. Educational Psychology (Dorchester-on-Thames). Abingdon: Taylor and Francis Group, 473–492.

[B32] Kizilenis Ulusman G. Turnalar-Çetinkaya N. Alpay Oraman E. (2023). “Compassion in organizations: A new perspective for maintaining diversity management,” in Academy of Management Proceedings. Academy of Management Briarcliff Manor, NY, 17808.30684404

[B33] Ko S. Choi Y. (2020). The effects of compassion experienced by SME employees on affective commitment: double-mediation of authenticity and positive emotion. Manage. Sci. Lett. 10, 1351–1358. 10.5267/j.msl.2019.11.022

[B34] Lilius J. M. Worline M. C. Dutton J. E. Kanov J. M. Maitlis S. (2011). Understanding compassion capability. Hum. Relat. 64, 873–899. 10.1177/0018726710396250

[B35] Lilius J. M. Worline M. C. Maitlis S. Kanov J. Dutton J. E. Frost P. (2008). The contours and consequences of compassion at work. J. Org. Behav. 29, 193–218. 10.1002/job.508

[B36] Maratos F. A. Gilbert P. Gilbert T. (2019). Improving Well-Being in Higher Education: Adopting a Compassionate Approach in Values of the University in a Time of Uncertainty. Cham: Springer, 261–278.

[B37] Marginson S. (2020). Public and Common Goods: Key Concepts in Mapping the Contributions of Higher Education. London: Bloomsbury Academic.

[B38] Mickey E. L. Misra J. Clark D. (2023). The persistence of neoliberal logics in faculty evaluations amidst COVID-19: Recalibrating toward equity. Gender Work Org. 30, 638–656. 10.1111/gwao.12817 35600799 PMC9111687

[B39] Nielsen M. W. Alegria S. Börjeson L. Etzkowitz H. Falk-Krzesinski H. J. Joshi A.. (2017). “Gender diversity leads to better science,” in Proceedings of the National Academy of Sciences - PNAS. National Academy of Sciences, 1740–1742. 10.1073/pnas.1700616114 PMC5338420 28228604

[B40] Nielsen M. W. Bloch C. W. Schiebinger L. (2018). Making gender diversity work for scientific discovery and innovation. Nat.re Hum. Behav. 2, 726–734. 10.1038/s41562-018-0433-1 31406295

[B41] Nishii L. H. (2013). The benefits of climate for inclusion for gender-diverse groups. Acad. Manage. J. 56, 1754–1774. 10.5465/amj.2009.0823

[B42] Nussbaum M. (1996). Compassion: the basic social emotion. Soc. Philos. Policy 13, 27–58. 10.1017/S0265052500001515

[B43] Østergaard C. R. Timmermans B. Kristinsson K. (2011). Does a different view create something new? The effect of employee diversity on innovation. Res. Policy 40, 500–509. 10.1016/j.respol.2010.11.004

[B44] Özbilgin M. (2009). Equality, Diversity and Inclusion at Work: Yesterday, Today and Tomorrow. London: Edward Elgar Cheltenham.

[B45] Özbilgin M. F. (2019). Global Diversity Management. Cham: Springer.

[B46] Özbilgin M. F. Erbil C. (2021). “Social movements and wellbeing in organizations from multilevel and intersectional perspectives: the case of the# blacklivesmatter movement,” in The SAGE Handbook of Organizational Wellbeing, ed. T. Wall (Cham: Springer), 119–138.

[B47] Özbilgin M. F. Erbil C. (2023). Insights into Equality, Diversity, and Inclusion. Contemporary Approaches in Equality, Diversity and Inclusion: Strategic and Technological Perspectives. Bingley: Emerald Publishing Limited, 1–18.

[B48] Pan S. (2020). COVID-19 and the Neo-Liberal Paradigm in Higher Education: Changing Landscape. Singapore: Asian Education and Development Studies.

[B49] Poorkavoos M. (2016). Compassionate Leadership: What is it and Why do Organisations Need More of It. Horsham: Roffey Park.

[B50] Radice H. (2013). How we got here: UK higher education under neoliberalism. ACME Int. J. Crit. Geograph. 12, 407–418.

[B51] Rapanta C. Botturi L. Goodyear P. Guàrdia L. Koole M. (2020). Online university teaching during and after the COVID-19 crisis: refocusing teacher presence and learning activity. Postdigital Sci. Educ. 2, 923–945. 10.1007/s42438-020-00155-y PMC7339092 40477148

[B52] Rynes S. L. Bartunek J. M. Dutton J. E. Margolis J. D. (2012). Care and Compassion Through an Organizational Lens: Opening up New Possibilities. Academy of Management Review. New York, NY: Academy of Management, 503–523.

[B53] Schein E. H. (2010). Organizational Culture and Leadership. New York, NY: John Wiley and Sons.

[B54] Shen P. Slater P. F. (2021). The effect of occupational stress and coping strategies on mental health and emotional well-being among university academic staff during the COVID-19 outbreak. Int. Educ. Stu. 14, 82–95. 10.5539/ies.v14n3p82

[B55] Simpson A. V. Clegg S. Pina e Cunha M. (2013). Expressing compassion in the face of crisis: Organizational practices in the aftermath of the brisbane floods of 2011. *J. Conting. Crisis Manag*. 21, 115–124. 10.1111/1468-5973.12016

[B56] Simpson A. V. Farr-Wharton B. (2017). The NEAR Organizational Compassion Scale: Validity, Reliability and Correlations. Wollongong, NSW: Australian and New Zealand Academy of Management.

[B57] Simpson A. V. Farr-Wharton B. (2017). The NEAR Organizational Compassion Scale: validity, reliability and correlations. Australian and New Zealand Academy of Management.

[B58] Simpson A. V. Farr-Wharton B. Cunha M. P. e Reddy P. (2019b). “Organizing organizational compassion subprocesses and mechanisms: A practical model,” in The Power of Compassion, eds. L. Galiana and N. Sansó (Nova Science Publishers, Inc), 339–357.

[B59] Simpson A. V. Farr-Wharton B. Reddy P. (2020). Cultivating organizational compassion in healthcare. J. Manage. Org. 26, 340–354. 10.1017/jmo.2019.54

[B60] Simpson A. V. Farr-Wharton B. S. R. Reddy P. (2019a). Correlating workplace compassion, psychological safety and bullying in the healthcare context. Acad. Manage. Proc. 10510:16632. 10.5465/AMBPP.2019.16632abstract

[B61] Simpson A. V. Pina e Cunha M. Rego A. (2015). Compassion in the context of capitalistic organizations: evidence from the 2011 Brisbane floods. J. Bus. Ethics 130, 683–703. 10.1007/s10551-014-2262-0

[B62] Simpson A. V. Rego A. Berti M. Clegg S. Cunha M. P. e. (2022). Theorizing compassionate leadership from the case of Jacinda Ardern: Legitimacy, paradox and resource conservation. Leadership, 18, 337–358. 10.1177/17427150211055291

[B63] Simpson A. V. Simpson T. Hendy J. (2023). Organising Compassionate Care with Compassionate Leadership in The Art and Science of Compassionate Care: A Practical Guide. Cham: Springer, 85–99.

[B64] Stellar J. E. Gordon A. M. Piff P. K. Cordaro D. Anderson C. L. Bai Y.. (2017). Self-transcendent emotions and their social functions: compassion, gratitude, and awe bind us to others through prosociality. Emot. Rev. 9, 200–207. 10.1177/1754073916684557

[B65] Strauss C. Taylor B. L. Gu J. Kuyken W. Baer R. Jones F.. (2016). What is compassion and how can we measure it? A review of definitions and measures. Clin. Psychol. Rev. 47, 15–27. 10.1016/j.cpr.2016.05.004 27267346

[B66] Urbina-Garcia A. (2020). What do we know about university academics' mental health? A systematic literature review. Stress Health 36, 563–585. 10.1002/smi.2956 32407607

[B67] Waddington K. (2016). The compassion gap in UK universities. Int. Prac. Dev. J. 6:10. 10.19043/ipdj.61.010

[B68] Waddington K. (2018). Developing compassionate academic leadership: The practice of kindness. J. Perspect. Appl. Acad. Pract. 87–89. 10.14297/jpaap.v6i3.375

[B69] Waddington K. (2021). Towards the Compassionate University: From Global Thread to Global Impact. New York, NY: Routledge.

[B70] Wallmark E. Safarzadeh K. Daukantaite D. Maddux R. E. (2013). Promoting altruism through meditation: an 8-week randomized controlled pilot study. Mindfulness 4, 223–234. 10.1007/s12671-012-0115-4

[B71] West M. A. (2019). “Compassionate leadership in health and care settings,” in The Power of Compassion, eds. L. Galiana and N. Sansó (Nova Science Publishers), 317–338.

[B72] West M. A. (2021). Compassionate Leadership: Sustaining Wisdom, Humanity and Presence in Health and Social Care. London: Swirling Leaf Press.

[B73] Wolbring G. Lillywhite A. (2021). Equity/equality, diversity, and inclusion (EDI) in universities: the case of disabled people. Societies 11:49. 10.3390/soc11020049

[B74] Wolbring G. Lillywhite A. (2023). Burnout through the lenses of equity/equality, diversity and inclusion and disabled people: a scoping review. Societies 13:131. 10.3390/soc13050131

[B75] Worline M. Dutton J. E. (2017). Awakening Compassion at Work: The Quiet Power That Elevates People and Organizations. Oakland, CA: Berrett-Koehler Publishers.

[B76] Worline M. C. Dutton J. E. (2022). The courage to teach with compassion: enriching classroom designs and practices to foster responsiveness to suffering. Manage. Learn. 22:13505076211044612. 10.1177/13505076211044611

[B77] Wray S. Kinman G. (2022). The challenges of COVID-19 for the well-being of academic staff. Occup. Med. 72, 2–3. 10.1093/occmed/kqab007 33585914 PMC7928699

